# Isolated amoebic brain abscess with excellent therapeutic response

**DOI:** 10.1590/0037-8682-0050-2022

**Published:** 2022-04-29

**Authors:** Tavares-Junior José Wagner Leonel, Arnaldo Ribeiro de Arruda, Pablo Picasso de Araújo Coimbra

**Affiliations:** 1 Universidade Federal do Ceará, Hospital Uniclinic, Fortaleza, CE, Brasil.; 2 Instituto Dr. José Frota, Hospital Uniclinic, Fortaleza, CE, Brasil.; 3 Uniclinic Diagnóstico por Imagem, Fortaleza, CE, Brasil.

Our patient was a previously healthy 40-year-old man. The patient presented with dysarthria and subtle monoparesis of the right upper limb. Brain magnetic resonance imaging (MRI) revealed a left frontal region contrast-enhancing lesion with associated edema (Figure A). He received albendazole (400 mg TID) for 30 days as treatment for neurocysticercosis; however, due to clinical deterioration, neurosurgery was performed with material drainage and biopsy. The lesion was still shown on the new brain MRI.

**FIGURE A:**
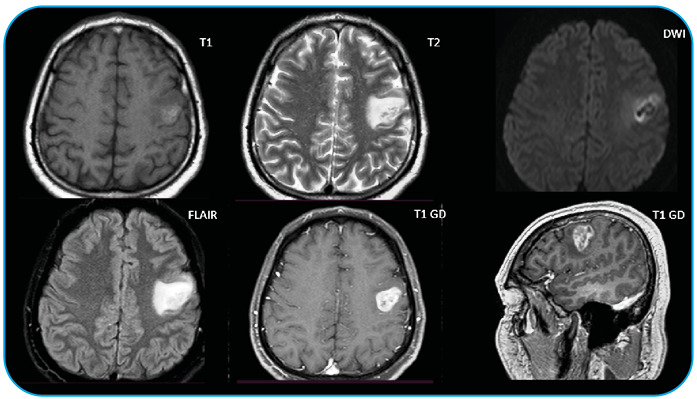
First MRI. Left to right, top to bottom, brain MRI sequences of axial slices in unenhanced T1, T2, diffusion, FLAIR, T1 with contrast, and sagittal T1 with contrast, respectively. Lesions pointed by blue arrows.

The histopathological results showed round lesions suggestive of trophozoites (Figure B). Immunohistochemistry with indirect immunoperoxidase yielded the presence of GFAP and macrophages CD68v, which indicated an infectious process. Tuberculosis, fungi, bacteria, cytomegalovirus, herpes simplex virus, and toxoplasma were ruled out using periodic acid-Schiff stain, Grocott methenamine-silver, and acid-fast bacillus (Figure B). The patient was eventually admitted in the hospital. Cerebrospinal fluid was collected from 2 cells and 17 proteins, and metronidazole (750 mg TID) and ceftriaxone (2 g BID) were administered as intravenous antibiotic therapy for four weeks. After completing the intravenous treatment, the patient received oral axetylcefuroxime (1 g BID) and metronidazole (750 mg TID) for another four weeks and underwent a repeat brain MRI, which showed radiological improvement (Figure C). After the treatment, the patient had no complaints or limitations. This report provides evidence of a favorable evolution. An amoebic brain abscess, caused by *Entameba histolytica* infection, is usually characterized by rapid evolution and high lethality if left untreated^1^. Similar cases have been described, but with unfavorable outcome^2^. This case report emphasizes the early diagnosis and treatment of suspected cerebral amebiasis cases^3^. 

**FIGURE B:**
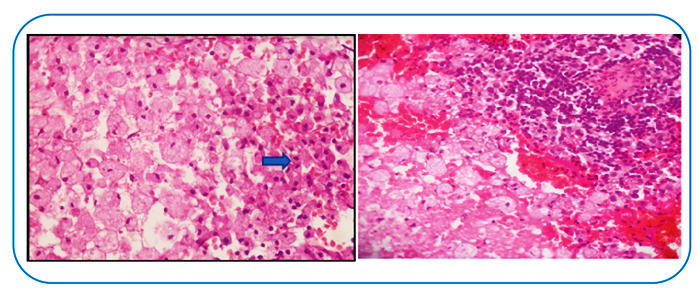
Focused photos. Sheets of xanthomized cells. On the left, 20× magnification; lymphocytic infiltrate (blue arrow), 40× magnification on the right.

**FIGURE C:**
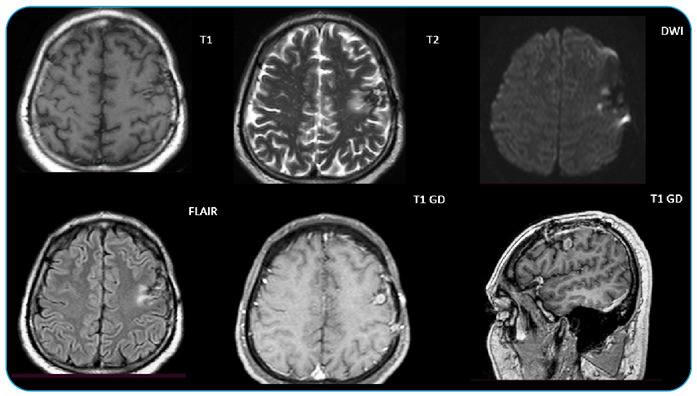
After the second month of amoebic brain abscess treatment. Left to right, top to bottom, brain MRI sequences of axial slices in unenhanced T1, T2, diffusion, FLAIR, T1 with contrast, and sagittal T1 with contrast, respectively. Radiological improvement after treatment demonstrated by blue arrows.
